# Role of miRNA in the regulation of cannabidiol-mediated apoptosis in neuroblastoma cells

**DOI:** 10.18632/oncotarget.26534

**Published:** 2019-01-01

**Authors:** Esraah Alharris, Narendra P. Singh, Prakash S. Nagarkatti, Mitzi Nagarkatti

**Affiliations:** ^1^ Department of Pathology, Microbiology and Immunology, University of South Carolina School of Medicine, Columbia, SC 29209, USA

**Keywords:** miRNA, cannabidiol, neuroblastoma, hsa-let-7a, hsa-miR-1972

## Abstract

Neuroblastoma (NBL) is one of the most common childhood cancers that originate from the immature nerve cells of the sympathetic system. Studies with NBL cancers have also shown that miRNAs are dysregulated and may play a critical role in pathogenesis. Cannabidiol (CBD) is a non-psychoactive compound found in marijuana which has been previously shown by our laboratory and others to induce apoptosis in cancer cells. However, there are no studies reported to test if CBD mediates these effects through regulation of miRNA. In the current study, therefore, we investigated if CBD induces apoptosis in human NBL cell lines, SH SY5Y and IMR-32, and if it is regulated by miRNA. Our data demonstrated that CBD induces apoptosis in NBL cells through activation of serotonin and vanilloid receptors. We also found that caspase-2 and -3 played an important role in the induction of apoptosis. CBD also significantly reduced NBL cell migration and invasion *in vitro*. Furthermore, CBD blocked mitochondrial respiration and caused a shift in metabolism towards glycolysis. CBD altered the expression of miRNA specifically, down-regulating hsa-let-7a and upregulating hsa-mir-1972. Downregulation of let-7a increased expression of target caspase-3, and growth arrest specific-7 (GAS-7) genes. Upregulation of hsa-mir-1972 caused decreased expression of BCL2L1 and SIRT2 genes. Together, our studies suggest that CBD-mediated apoptosis in NBL cells is regulated by miRNA.

## INTRODUCTION

Neuroblastoma (NBL) is a tumor characterized by heterogeneity and variable clinical outcomes arising from premature sympathetic neurons, and is most commonly encountered in infants and young children [1]. According to the American Cancer Society, the 5-year survival rate for NBL is less than 50% after treatment with surgery followed by chemotherapy or radiotherapy. These outcomes are likely due to both the relapse of tumor and the cytotoxic side effects of intensive therapy. Although variable treatment strategies have been established and have been effective in treating the tumor, post-intervention rates remain constant due to side effects of treatment [2].

Cannabidiol (CBD) is a member of a group of compounds known as cannabinoids that are found in the plant, *Cannabis sativa* [3]. Cannabinoids act primarily through activation of CB1 or CB2 receptors [4]. Both receptors are G-protein coupled receptor but the CB1 receptors are predominantly expressed in the neurons whereas the CB2 receptors are mainly located in the immune cells [3]. CBD is free of psychoactive effect because it doesn’t have a significant affinity for both receptors [5].

Our laboratory was one of the first ones to demonstrate that cannabinoids can induce apoptosis in cancer cells and when injected into mice, could cause syngeneic tumor rejection [6]. Since this seminal observation, a large number of publications have confirmed and extended these studies to a variety of tumors that express cannabinoid receptors. Interestingly, we and others have shown that CBD can also induce apoptosis in many types of cancers such as breast, glioma, glioblastoma, and leukemia [7–11]. While different signaling pathways have been identified that trigger apoptosis in cancer cells following treatment with CBD, whether such events are mediated by microRNA (miRNA) has not been previously investigated.

miRNAs are small non-coding RNAs which are involved in RNA silencing and post-transcriptional regulation of gene expression. MiRNAs play a key role in cancer biology and help determine the nature of the tumor, prognosis and response to treatment. The first report on role of miRNA in cancer was suggested by identifying miR-15a/16-1 cluster deletion in human chronic lymphocytic leukemia [12]. This deletion induced overexpression of the anti-apoptotic B-cell lymphoma 2 (BCL2), which was a target of these miRNAs [12]. Specifically, studies with NBL cancers have also shown that miRNAs are dysregulated and may play a critical role in the pathogenesis. For example, the *miRNA-17-5p-92* cluster was over-expressed in NB cells lines exhibiting overexpression of *MYCN* [13]. Interestingly, *in vitro* or *in vivo* treatment of MYCN-amplified and therapy-resistant neuroblastoma cells with antagomir-17-5p led to inhibition of growth of these cancer cells through activation of apoptosis [13]. In addition, MYCN has been shown to be regulated by histone deacetylases (HDAC) such as HDAC5 and SIRT2 [14, 15]. MiRNA dysregulation has also been associated with development of resistance to therapies. For example, during the development of resistance, cancer cells expressed decreased levels of miRNAs, such as miRNA-200c and miRNA-579-3p, two potent oncosuppressors [16, 17]. Thus, restoration of their expression led to increased efficacy of drugs that targeted MAPK pathway.

We previously showed that CBD can induce apoptosis in human leukemic cells *in vitro* and when injected into mice, cause syngeneic tumor regression [11]. In this model, treatment of cancer cells with CBD increased the levels of reactive oxygen species (ROS) and NAD (P)H oxidases Nox4 and p22(phox), while causing a decrease in the levels of p-p38 mitogen-activated protein kinase [11]. Other studies have also shown that CBD induces apoptosis via inhibition of Akt/mTOR pathway [18] and this relates to the fact that Akt is overexpressed in many human cancers and is responsible for their resistance to apoptosis [19]. Despite such studies, no previous studies have explored the role of miRNA in CBD-mediated induction of apoptosis in cancer cells. To that end, in the current study we identified miRNA that are modulated by CBD and studied their potential role in inducing apoptosis in NBL cells.

## RESULTS

### CBD induces apoptosis in NBL cell lines, SH SY5Y and IMR-32, through activation of caspase-2 and caspase-3

**Figure 1:**
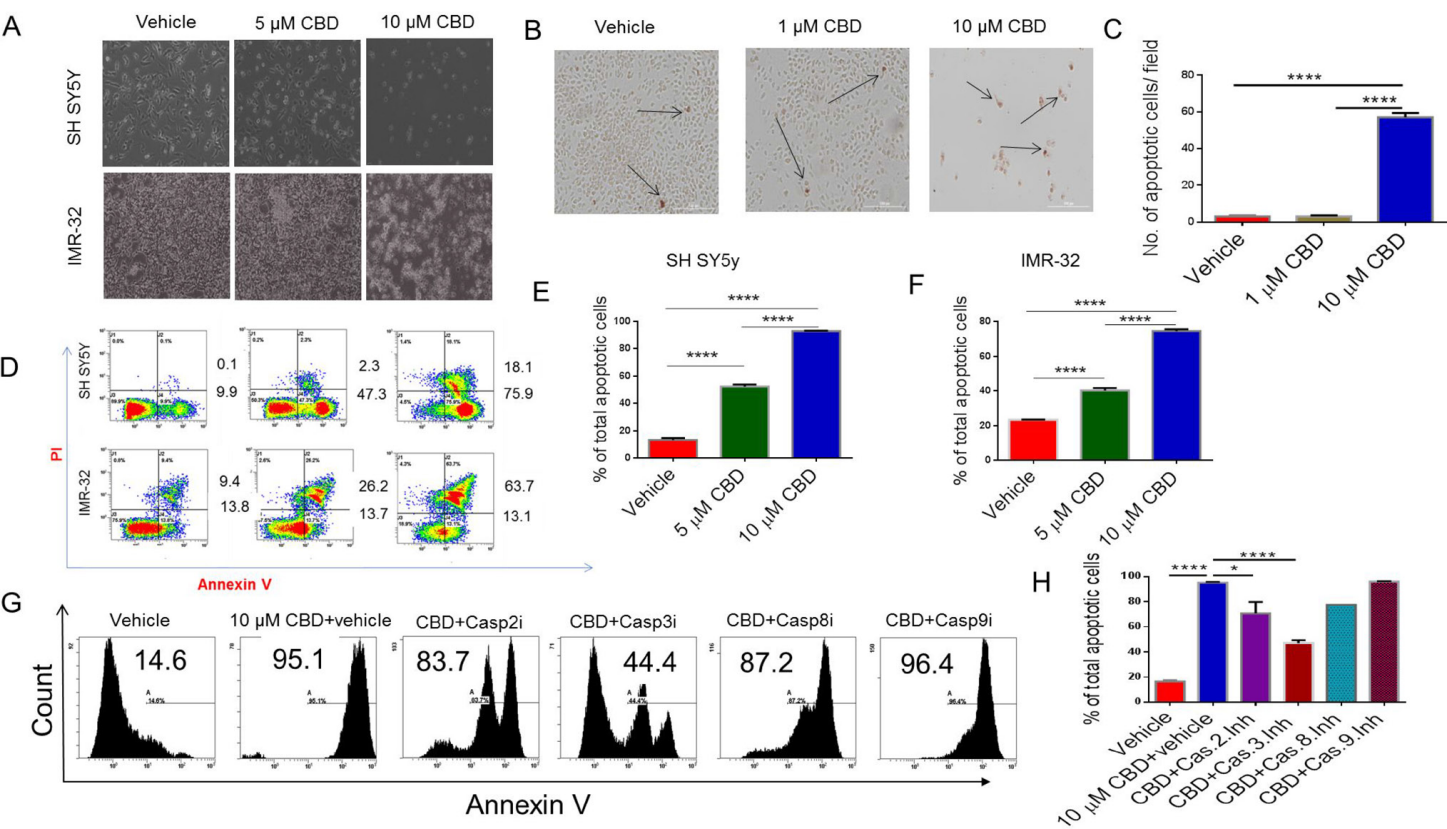
Treatment with CBD induces apoptosis in neuroblastoma cell lines SH SY5Y and IMR-32 neuroblastoma cell lines were treated with vehicle or CBD in serum-free medium for 24 hours. (**A**) Bright field image showing that CBD induces significant morphological damage in neuroblastoma cells in a dose-dependent manner. (**B**) Tunnel assay for SH SY5Y after treatment with either vehicle or CBD (1 or 10 µM). The arrows are pointing to the nuclei of the dead cells which are stained dark brown with HRP-labeled streptavidin. (**C**) Bar diagram shows the number of apoptotic cells per field in B. (**D**) Flow cytometry analysis of SH SY5Y and IMR-32 cell lines after 24 hours treatment with either vehicle or CBD (5 or 10 µM). Cells in early apoptosis stain with Annexin-V only while those in late apoptosis are double-stained with Annexin-V and PI. Panels (**E**) and (**F**) show the statistical analysis of data from multiple experiments for SH SY5Y and IMR-32 cell lines, respectively as detailed in panel D. (**G**) Representative experiment in which SH SY5Y cells were treated with vehicle or 10 µM CBD+vehicle or CBD+caspase inhibitors and cells analyzed for apoptosis using Annexin V staining. (**H**) Data from panel G in multiple experiments plotted as Mean ± SEM. All experiments were repeated three times. Significance (*p* < 0.05) for all experiments was calculated using one-way ANOVA and post-hoc Tukey’s test.

To examine the morphological effects of CBD on SH SY5Y and IMR-32 NBL cell line, we visualized them by bright field microscopy at 20× magnification. Apoptotic signs were assessed for clumping, blebbing, and shrinking. In contrast to the vehicle group, CBD-treated cells displayed elevated apoptotic rates (Figure 1A). DeadEnd Colormetric TUNEL assay showed a significant increase in the number of positively stained (brown) cells in 10 µM CBD-treated cells when compared to the vehicle CBD-treated groups; *p* < 0.001 (Figure 1B and 1C). Flow cytometry analysis of SH SY5Y and IMR-32 showed a significant increase in the number of the cells stained with AnnexinV (early apoptosis) and both Annexin-V and PI (late apoptosis) in 5 and 10 group when compared to vehicle controls (Figure 1D). Data from multiple flow cytometric analyses similar to that presented in Figure 1D have been expressed as Mean ± SEM of total (early+late) apoptotic cells in Figure 1E and 1F panels.Also, 10 µM CBD caused significantly higher apoptosis when compared to 5 µM CBD treated cells (Figure 1D–1F). Flow cytometry analysis was performed to investigate which caspases mediate CBD-induced apoptosis. Annexin-V-PI staining showed significant reduction in apoptosis in cells incubated with CBD and pre-treated with caspase-2 and caspase-3 inhibitors when compared to CBD+vehicle controls (Figure 1G, 1H) In contrast, Caspase 8 and 9 inhibitors failed to cause significant inhibition in apoptosis.

### Identification of receptors through which CBD induces apoptosis

**Figure 2:**
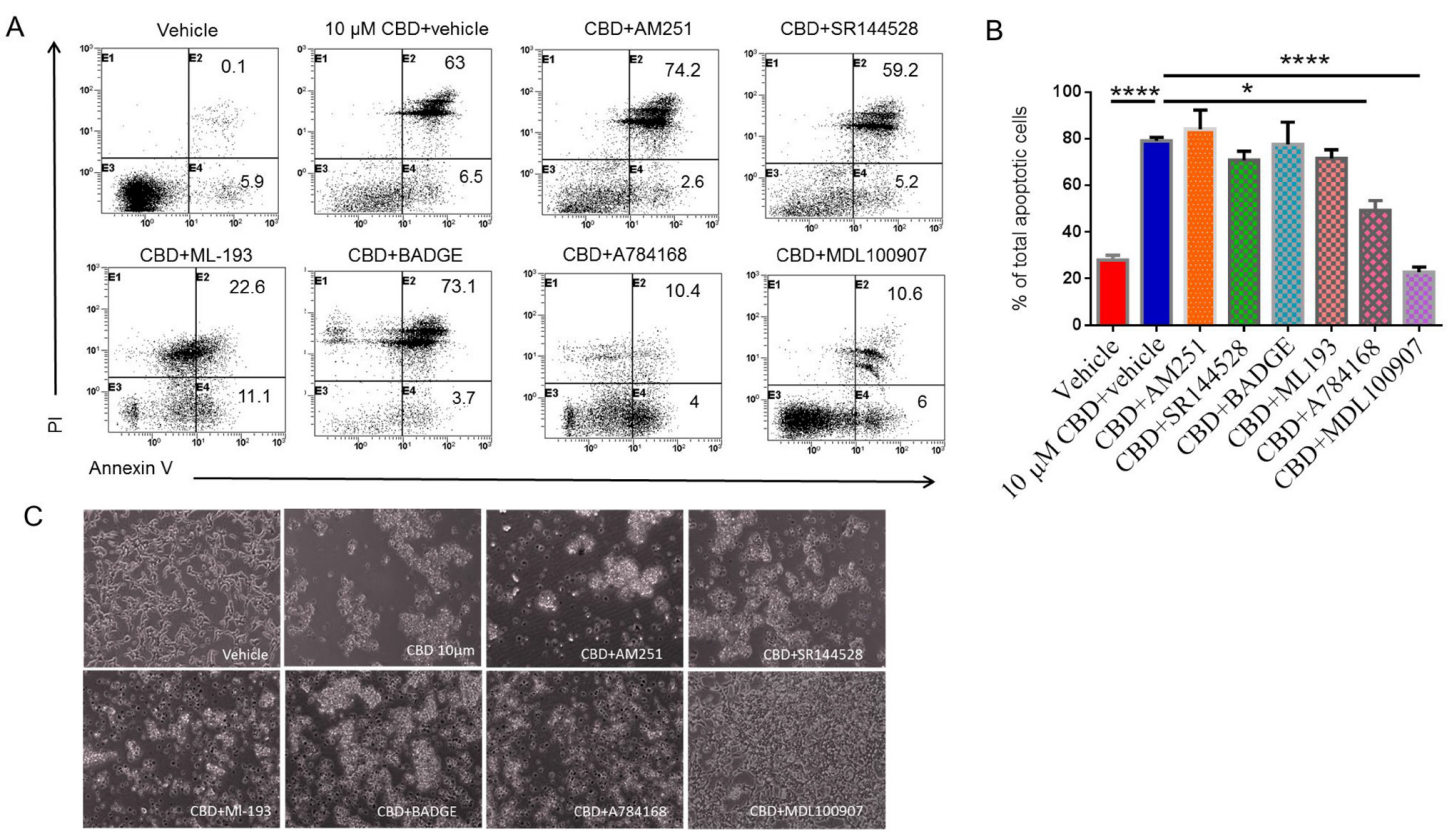
Identifying the receptors through which CBD induces apoptosis in SH SY5Y neuroblastoma cells SH SY5Y cells were plated overnight and then treated either with vehicle or receptor antagonist for 1 hour followed by 10 µM CBD. Next, the cells were harvested and stained with Annexin-V- PI followed by flow cytometric analysis. The data from a representative experiment has been shown (**A**) and data from multiple experiments have been plotted in the bar diagram (**B**). (**C**) shows morphology of cells exposed to cultures as described in Panel A. Significance (*p* < 0.05) for all experiments was determined using one-way ANOVA and post-hoc Tukey’s test.

To determine which receptor plays a role in CBD-induced apoptosis, we utilized several receptor antagonists followed by staining and flow cytometric analysis of apoptotic cells. A representative flow experimental data has been presented in Figure 2A with cells stained for AnnexinV only (early apoptosis) and for both Annexin-V and PI (late apoptosis). Also, data from multiple experiments have been shown as Mean ± SEM of total (early+late) apoptotic cells in Figure 2B. Panel C shows morphology of cells exposed to various cultures conditions. There was a significant reduction in the percentage of total apoptotic cells induced by CBD in samples pre-treated with GPR55 antagonist (ML-193), TRPV1 antagonist (A784168), or 5-HT2A receptor antagonist (MDL100907) when compared to CBD+vehicle group (Figure 2A–2C). However, CB1 antagonist (AM251), CB2 antagonist (SR144528), GPR-55 (ML-193), or PPAR-γ antagonist (BADGE), failed to inhibit CBD-mediated apoptosis. These data suggested that CBD may induce apoptosis through activation of 5-HT2A and TRPV1 receptors.

### Role of miRNA in the regulation of CBD-mediated apoptosis

**Figure 3:**
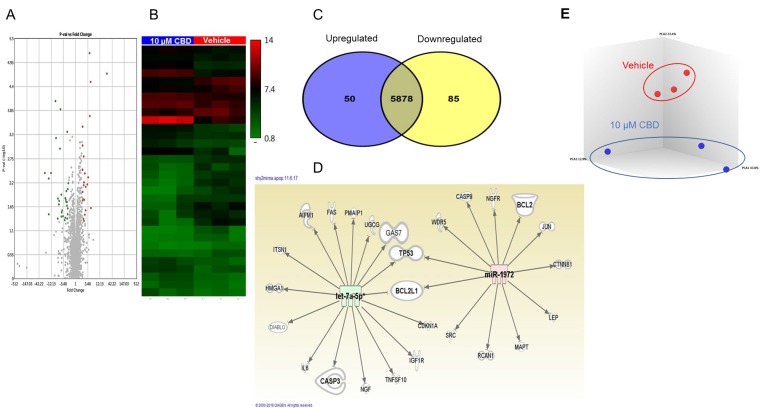
miRNA profile in SH SY5Y neuroblastoma cells following CBD treatment Microarray analysis was done by using an Affymetrix array to identify miRNA in CBD-treated cells compared to vehicle controls. (**A**) Volcano plot for up- and down-regulated miRNAs following treatment either with DMSO (vehicle) or 10 µM CBD (CBD). The upregulated miRNAs appear in red color while the down-regulated are shown in green dots using TAC software from Affymetrix. (**B**) Heat map for dysregulated miRNA following treatment with CBD (TAC software). The red color represents the upregulated miRNAs while the blue color represents the downregulated ones. (**C**) Venn diagram shows differentially regulated miRNA in the two groups. (**D**) Ingenuity Pathway Analysis displaying the relationship between the dysregulated miRNA and different target genes related to apoptosis. The panel shows how miRNA hsa-let-7a (green) and hsa-miRNA-1972 (red) may target apoptotic pathways. (**E**) Principal Component Analysis of 3 independent samples showing distinct clustering of miRNA profiles in vehicle- and CBD-treated groups.

We performed miRNA array in CBD-treated SH SY5Y cells to elucidate the role of miRNA in CBD-induced apoptosis. Volcano plots and heat map showed that CBD treatment induced changes in the miRNA profile (Figure 3A and 3B). A Venn diagram demonstrated that SH SY5Y cells treated with 10 µM CBD induced upregulation of 50 miRNAs and downregulation of 85 miRNAs (Figure 3C). The dysregulated miRNAs with fold change ≥2 or ≤–2 were uploaded to Ingenuity Pathway Analysis (IPA) from Qiagen. IPA software showed that hsa-let-7a was highly downregulated (-12 fold) and was targeting caspase-3, GAS-7, and DIABLO genes (Figure 3D). In contrast, CBD treatment caused upregulation of hsa-miRNA-1972 (>2 fold) that targeted BCL2 and BCL2L1 genes (anti-apoptotic mitochondrial proteins). Principal Component Analysis of 3 independent samples showed distinct clustering of miRNA profiles in vehicle- and CBD-treated groups (Figure 3E). Because down-regulation of the expression of miRNA leads to induction of the target gene while its upregulation leads to gene silencing, our data suggested that the changes in miRNA induced by CBD may collectively promote apoptosis.

### CBD induced alterations in miRNA expression and their effect on target genes involved in apoptosis

**Figure 4:**
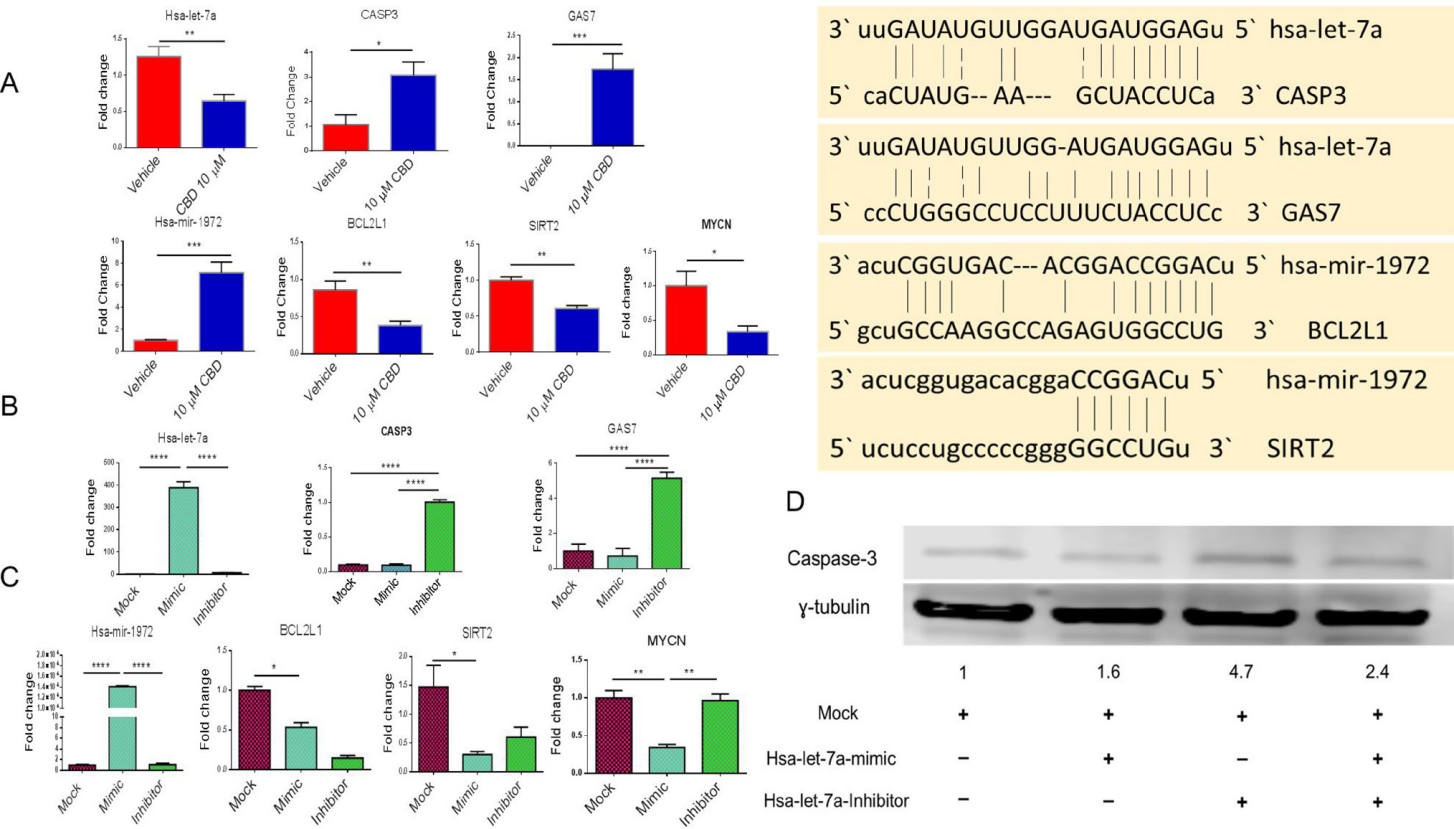
Validation of miRNA and their potential targets (**A**) SHSY 5Y cells were cultured with either DMSO or 10 µM CBD as described in Figure 1 legend. Next, qPCR was performed for the genes of interest according to Ingenuity Pathway Analysis map. (**B**) SH SY5Y cells were treated with either mock, hsa-let-7a-mimic or hsa-let-7a inhibitor for 24 hours. Next, qPCR was performed. We validated that hsa-let-7a transfection was successful as shown in the first panel. The next two panels show the levels of expression of caspase-3 and GAS7. (**C**) SH SY5Y cells were treated with mock, hsa-miRNA-1972 mimic or hsa-miRNA-1972 inhibitor. Next, qPCR was performed. First panel shows validation that hsa-miRNA-1972 transfection was successful. The next three panels show the levels of expression of BCL2L1, Sirt-2, and MYCN. (**D**) After transfecting SH SY5Y with hsa-let-7a mimic and inhibitor, protein was obtained and western blot was done to detect the level of caspase-3. The level of caspase-3 protein was 4.7 folds higher than the mock control. Significance (*p* < 0.05) for Panel A was performed by using Student’s *t*-test and for Panel B and C was determined using one-way ANOVA and post-hoc Tukey’s test.

We performed qRT-PCR for the validation of miRNA and genes identified by IPA software. Gene alignment software predicted that miRNA-hsa-let-7a targeted Caspase-3 and GAS-7 genes while miRNA-hsa-1972 targeted BCL2 and SIRT2 genes (Figure 4A). We found that CBD treatment of cells led to significant downregulation of miRNA-hsa-let-7a with consequent increase in Caspase-3 and GAS-7 (Figure 4A). On the other hand, hsa-miRNA-1972 was significantly upregulated in CBD-treated group when compared to the vehicle, and the related genes, BCL2L1, SIRT2 and MYCN were significantly downregulated in CBD-treated group versus vehicle (Figure 4A). While most of the miRNA regulated molecules that we studied were associated with apoptosis, we did include other molecules such as SIRT2 because SIRT2 was shown to be upregulated by N-Myc in neuroblastoma cells and that SIRT2 promoted cancer cell proliferation [14]. Also, GAS-7 is involved in controlling growth arrest and apoptosis of neuroblastoma cells in response to various stimuli [20].

To further validate the role of miRNA in the regulation of aforesaid target genes, we performed transfections of hsa-let-7a mimic or inhibitor in NBL cell line. In this experiment, we found that transfection of hsa-let-7a inhibitor significantly induced caspase-3 and GAS-7 expression when compared to mock control (Figure 4B). Transfection of a hsa-miRNA-1972 mimic caused significant downregulation of BCL2L1, MYCN and SIRT2 when compared to mock control (Figure 4C). Protein levels of caspase-3 using western blotting shows that the expression of caspase-3 in the cells transfected with hsa-let-7a inhibitor was 4.7 folds higher than the cells transfected with the mock control (Figure 4D).

### CBD targets genes involved in cell migration, invasion, metabolism and apoptosis

**Figure 5:**
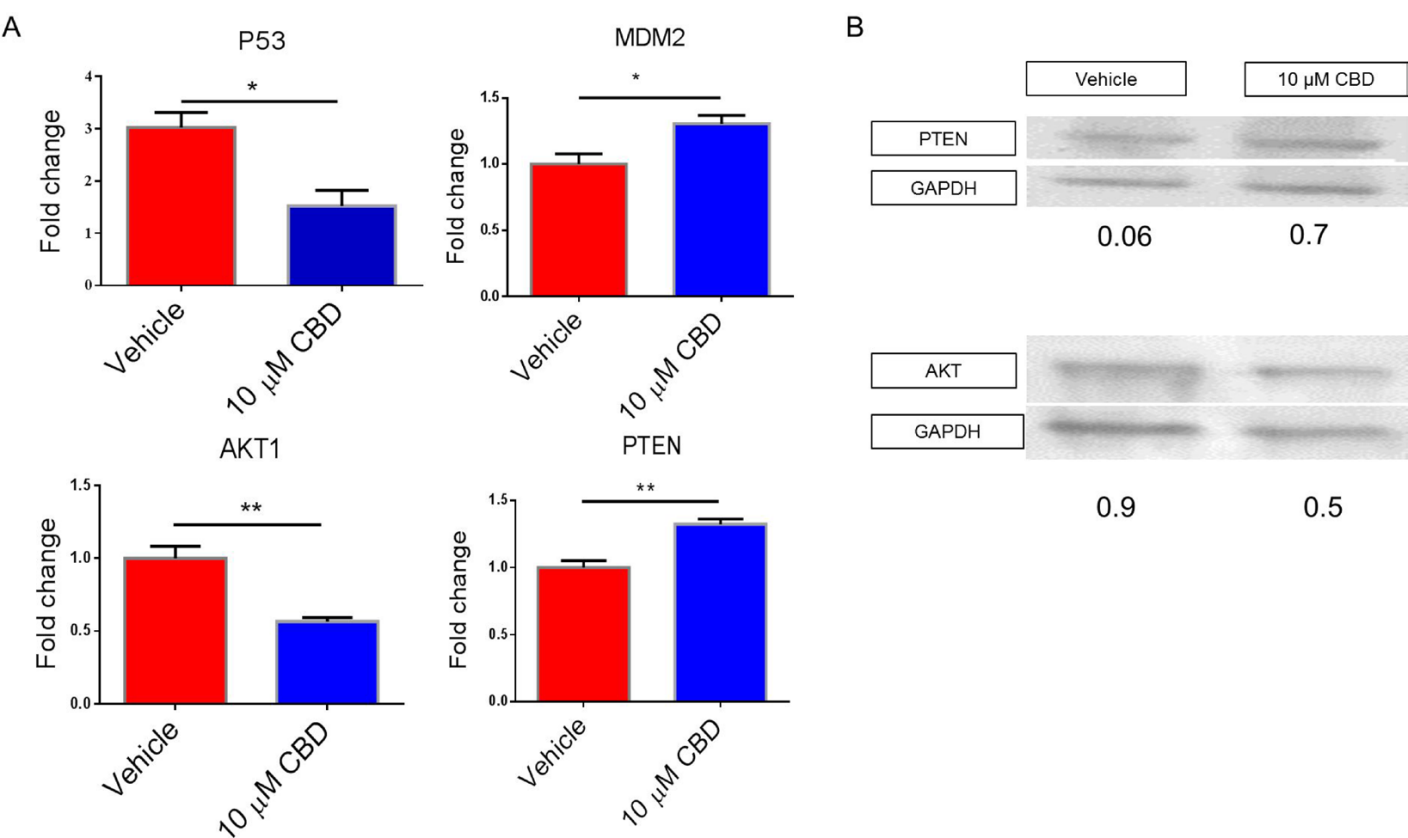
Effect of CBD on target genes involved in cell migration, invasion, metabolism and apoptosis SH SY5Y cells treated with vehicle or 10 µM CBD were used to perform (**A**) qPCR or (**B**) Western Blots for the genes of interest according to Ingenuity Pathway Analysis map. In Panel A, significance (*p* < 0.05) was determined using Student’s *t*–test.

We examined other potential genes that are regulated by altered expression of miRNA and found that P53 and AKT1 expression was significantly downregulated while the expression of MDM2 and PTEN was significantly higher in the CBD treated group when compared to vehicle (Figure 5A). SDS-PAGE and subsequent western blot were performed to validate gene expression data. Our analysis on whole cell lysate demonstrated the CBD-treated NBL cell line showed decreased AKT and increased PTEN proteins when compared to the vehicle control (Figure 5B).

### CBD inhibits cell migration, invasion and mitochondrial respiration of NBL cells

**Figure 6:**
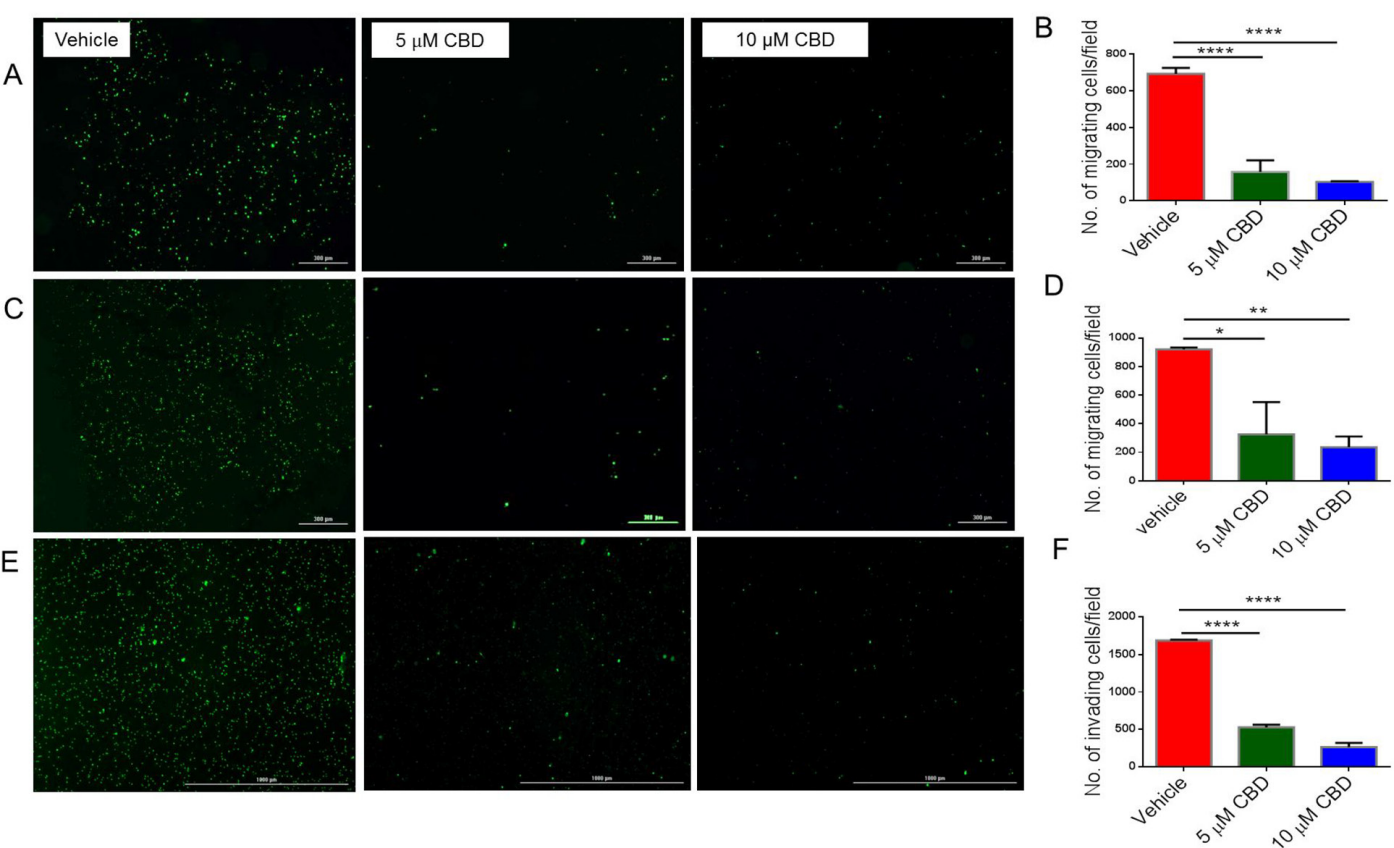
CBD inhibits cell migration, invasion and mitochondrial respiration of neuroblastoma cells (**A**) SH SY5Y cells (**C**) IMR-32 cells were cultured with vehicle, 5 µM, or 10 µM CBD and stained with Vybrant CFDA. Live cells were seeded per floroblock inserts of a 24-well plate. Cells were allowed to migrate for 24-hour. Next, cells were visualized using Cytation 5 microscope. Panel A and C show data from a representative experiment while (Panel **B** and **D**) show data from multiple experiments, respectively. (**E**) SH SY5Y cells were cultured as in Panel A with vehicle, 5 µM, or 10 µM CBD and live cells were seeded in matrigel-coated inserts for 24-well plate. Cells were allowed to migrate for 24-hour. Then cells were visualized using Cytation 5 microscope. Panel E shows data from a representative experiment while (Panel **F**) shows data from multiple experiments. Significance (*p* < 0.05) for all experiments was determined using one-way ANOVA and post-hoc Tukey’s test. Each experiment was repeated three times.

**Figure 7:**
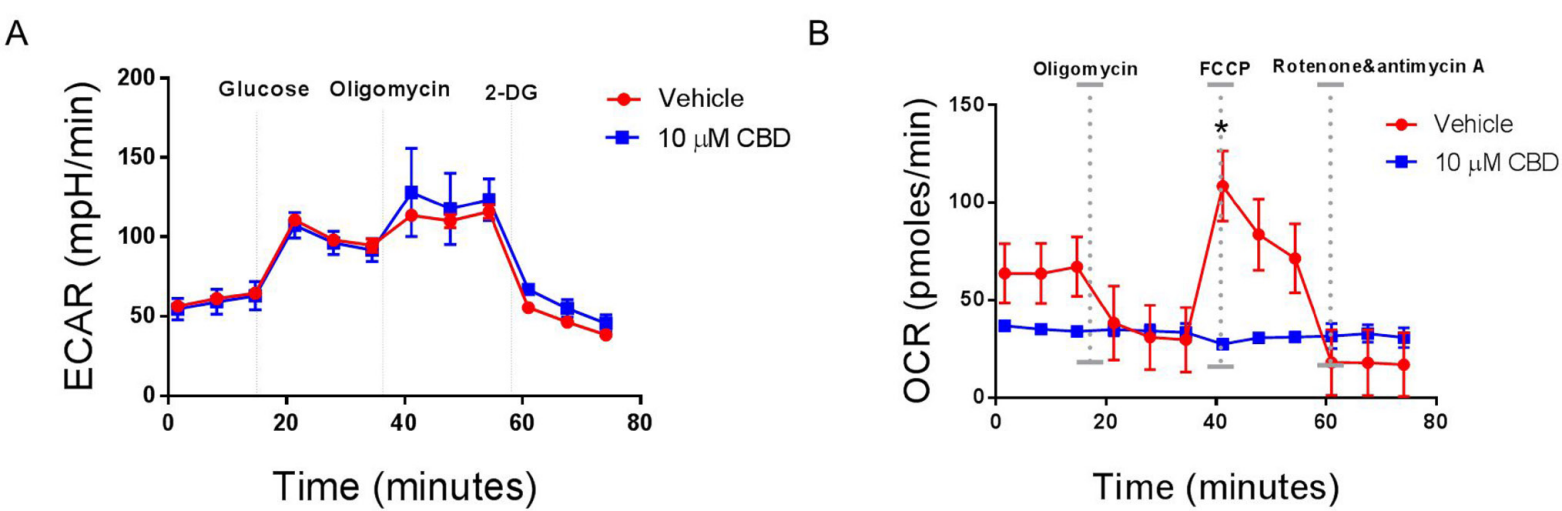
CBD alters mitochondrial respiration in neuroblastoma cells SH SY5Y cells were cultured overnight in a Sea-horse Analyzer plate. The following day, the cells were treated with either vehicle or 10 µM CBD, washed with Seahorse-specific medium, and placed in a Seahorse analyzer. (**A**) Analysis of glycolysis by ECAR determination. (**B**) Analysis of mitochondrial respiration by OCR analysis. Significance (*p* < 0.05) for all experiments was done using Student’s *t*-test. Each experiment was repeated three times.

One important feature of recurrent malignant diseases is their ability to migrate, invade, and shift their metabolism. For this reason, we measured the ability of CBD to inhibit cancer migration, invasion and metabolism. We found that CBD was able to significantly inhibit SH SY5Y (Figure 6A and 6B), IMR-32 cell migration (Figure 6C and 6D) and invasion through matrigel (Figure 6E and 6F). Additionally, CBD altered mitochondrial respiration, particularly maximal respiration, measured via oxygen consumption rate (OCR), which was significantly reduced after CBD treatment (Figure 7B). On the other hand, extracellular acidification rate (ECAR) that measures the rate of glycolysis, did not change (Figure 7A). As a result, we believe that CBD treatment inhibited the mitochondrial respiration and shifted the cell metabolism towards the glycolysis pathway.

## DISCUSSION

Our laboratory was one of the first one to discover that cannabnoids can induce apoptosis in cancer cells through activation of cannabinoid receptors [6]. This has led to additional studies on the effect of cannabinoids in the treatment of cancer [21–28]. CBD is a non-psychoactive cannabinoid that has been reported to have the potential efficacy to treat breast, prostate, glioma, lung and cervical cancers [8, 9, 27, 29]. Neuroblastoma (NBL) constitutes one of the most common solid cancers in children. While chemo, radiation, and immunotherapy are used to treat NBL, children with this cancer have poor outcomes. Also, while CBD has been shown to kill NBL cells [21], the precise molecular pathways remain further elucidation. Importantly, there are no previous studies delineating the role of miRNA in the anti-cancer properties of CBD. Such studies are important because they will also help identify additional miRNA targets that can be used to treat NBL.

In the current study, we used serum-free medium to investigate the effect of CBD on SH SY5Y and IMR-32 NBL cell lines. This was because fetal bovine serum contains endocannabinoids which can interfere with the action of CBD on various receptors [30, 31]. It was for this reason that in our previous studies as well, we used serum-free medium to test the ability of CBD to induce apoptosis in Jurkat human leukemic cells [11]. In the current study, we used caspase inhibitors (caspase-2, -3, -8, and -9) to explore the role of caspases and found that apoptosis in NBL cells was dependent on caspase-2 and 3 but not 8 and 9. Caspase 8 is known to regulate death cell receptor (extrinsic) pathway, while Caspase-9 controls the mitochondrial pathway [32]. Interestingly, when we looked at the role of Caspase-2, which is similar to caspase-9 in structure, we found that inhibition of Caspase-2 attenuated CBD-induced apoptosis. This finding is consistent with previous studies in which it was shown that Caspase-2 may play a key role in apoptosis induced by metabolic imbalance, DNA damage, and endoplasmic reticulum (ER) stress [33]. Previous studies have shown that apoptosis induced by indoles is also mediated by Caspase-2 but independent of Caspase-8 and -9 [34]. Caspase-2 is one of the most evolutionarily conserved caspases, and whether or not Caspase-2 fits the role of a traditional initiator or effector caspase, or both, remains to be established. However, Caspase-2 can engage the mitochondrial pathway to trigger apoptosis involving caspase-independent death effectors apoptosis-inducing factor (AIF) and endonuclease G [35] Such a mechanism can explain how CBD can engage Caspase-2 in the induction of the mitochondrial pathway, independent of caspase-9. Caspase-2 is also the sole caspase known to translocate from the cytosol to the nucleus, thereby suggesting that it is involved in cellular processes other than apoptosis [36].

CBD does not have much affinity towards CB1 and CB2 receptors because of which it is not psychoactive. In the current study, we also noted that CBD-mediated apoptosis was not blocked by CB1 and CB2 antagonists. However, we noted that CBD may induce apoptosis through activation of 5-HT2A and TRPV1 receptors, based on blocking studies. CBD is well established to bind and function primarily through activation of TRPV1 or vanilloid receptors [37]. Researchers have also documented that CBD uses vanilloid and 5-hydroxy tryptamine receptors to induce autophagy or prevent cancer growth [23–25]. Activation of the TRPV1 receptor has been shown to increase Ca^++^ in the cytoplasm followed by ER stress and apoptosis in gliomas [38] and in prostate cancer [39]. GPR-55 is an orphan G-protein coupled receptor expressed in the brain and in cell cultures, and is known to bind to certain cannabinoid ligands [40]. However, the role of GPR-55 in CBD functions remains unclear. In an earlier study, we found that CBD could induce apoptosis in human leukemic cells. CBD increased reactive oxygen species (ROS) production as well as NAD(P)H oxidases Nox4 and p22(phox) [11]. CBD also caused a decrease in the levels of p-p38 mitogen-activated protein kinase, which could be blocked by treatment with a CB2-selective antagonist or ROS scavenger [11]. These data and the current study suggested that CBD-mediated apoptosis in cancer cells may involve different pathways and receptors.

Gene expression is regulated by different mechanisms including miRNA regulation, which constitute small protein non-coding, 20–25 nucleotides long [41]. Comparisons of miRNA expression in malignant and normal cells highlight the importance of cancer-related miRNAs. Additionally, miRNAs may function as oncogenes or tumor-suppressor genes [42]. Previous studies have shown that miRNAs have a critical role in cell growth, differentiation, and apoptosis [43]. We found that let-7a was significantly down-regulated in CBD treated cells versus vehicle. It has been shown that let-7a is a tumor suppressor in colon [44] and prostate cancer [45]. Other studies reported that let-7a targets caspase-3 and thus, down-regulation of let-7a increases drug-induced apoptosis [46]. In the current study, we found that downregulation of hsa-let-7a could upregulate caspase-3 gene expression in CBD-treated SH SY5Y cells. Furthermore, hsa-let-7a inhibition led to induction of GAS-7, a member of protein family known as PCH (Pombe Cdc 15 homology) which is mainly localized in neurons and is responsible for differentiation and survival [47, 48]. The potential role of GAS-7 in cancer or apoptosis remains to be determined. In addition to let-7a downregulation, we found that CBD caused upregulation of hsa-miRNA-1972, which based on gene alignment scores, was predicted to bind Sirt-2 and BCL2L1 genes. Downregulation of Sirt-2 reduces MYCN gene expression and results in apoptosis in a neuroblastoma cell line [14, 15]. Therefore, we propose that upregulation of hsa-miRNA-1972 resulted in the downregulation of both genes and induced death in NBL cells. Both genes have been extensively studied in cancer and have shown to be regulated by several miRNA [49–52]. To confirm our finding that CBD caused a shift in miRNA expression, we transfected let-7a mimic and inhibitor into NBL cell lines. Our data showed that gene expression of CASP3 and GAS-7 was significantly higher in the cells transfected with hsa-let-7a-inhibitor when compared to those transfected with let-7a mimic. In addition, caspase-3 protein expression was higher in SH SY5Y cells transfected with hsa-let-7a inhibitor as compared to those transfected with mock control.

The reduced expression of MYCN and P53 genes (the latter could be as a result of MDM2 gene upregulation since it is the negative regulator of p53 could play an important role in reducing NBL cells survival [53, 54]. Also, PTEN tumor suppressor protein inhibits activation of Akt [55], which restricts MDM2 to the cytoplasm. Associations between MYCN and p53 were recently confirmed [56]. Our western blot analysis showed that the protein expression of PTEN was significantly upregulated in the CBD-treated group while that of Akt was significantly downregulated when compared to the vehicle. We also found that following CBD treatment, there was significant inhibition of SH SY5Y cell migration and invasion, and this may be mediated through AKT-signaling. PTEN activity has been shown to inhibit cell migration and invasion [57–59]. Additionally, PTEN may play a major role in apoptosis as well by inhibiting AKT signaling [60–62]. Our findings suggest that CBD induces apoptosis in NBL cell line by inhibition of Akt protein mediated by PTEN upregulation.

In the current study, we also found that CBD inhibited cell migration, invasion and caused alterations in metabolism. This is consistent with previous studies demonstrating that cannabinoids were able to inhibit cancer migration, invasion and metabolism in various cancers [28, 63, 64]. Seahorse XFp analysis for SH SY5Y cells detected a shift towards glycolysis to skip the effects of CBD on their metabolism, an effect known as Warburg effect [65]. The Warburg effect is the ability of cancer cells to shift the generation of ATP from oxidative phosphorylation to glycolysis and can be regulated by the AKT/mTOR pathway [66, 67]. Our data suggested the metabolic dysfunction in CBD-treated cells is through the AKT-dependent mechanism. In addition, downregulation of MYCN expression has been shown to be responsible for shutting down glycolysis [68]. Early response of SH SY5Y by significant reduction of mitochondrial respiration has been noted before with other neurotoxins [69].

There is growing evidence that miRNAs can regulate glycolysis directly by regulating the expression of genes encoding for glycolysis pathway or indirectly by controlling the expression of oncogenes and tumor suppressor genes involved in glycolysis. Hsa-let-7a has been shown to regulate glycolysis in various cancers [70–72]. In addition, SIRT2, which was downregulated by hsa-miRNA-1972 has been shown to be a potent regulator of glycolysis [73]. Thus, the alterations caused by CBD in miRNA may also be responsible for changes in cell metabolism. Together, the current study suggests that CBD alters the expression of several miRNA that target critical signaling pathways implicated in apoptosis, migration and invasion, and metabolic functions in NBL cells.

## MATERIALS AND METHODS

### Cell lines and reagents

Human NBL cell lines SH SY5Y and IMR-32 were purchased from ATCC (American Type Culture Collection, Manassas VA) and were grown in DMEM; Dulbecco’s Modified Eagle’s Medium (ThermoFisher Life Technologies, Grand Island, NY, USA) supplemented with 10% heat-inactivated fetal bovine serum (Atlanta Biologicals, Lawrenceville, GA), 100 units/ml penicillin, 100 μg/ml streptomycin, in 5 mM Glutamine (ThermoFisher Life Technologies, Grand Island, NY, USA). Cell cultures were maintained in a humidified incubator set to 37° C and 5% CO_2_. CBD was purchased from Cayman Chemicals, reconstituted in DMSO at a concentration of 20 mg/ml, aliquoted and stored in –20° C.

Immediately before adding CBD to cells, it was diluted in serum-free DMEM at a concentration of 5 and 10 µM. The vehicle group received DMSO at the same dilution. Caspase inhibitors: caspase-2 (Z-VDVAD-FMK-cat.no.FMK003), caspase-3 (Z-DEVD-FMK-cat.no. FMK004), caspase-8 (Z-IETD-FMK-cat.no.FMK007), and caspase-9(Z-LEHD-FMK-cat.no.FMK008) were purchased from R&D system (Minneapolis, MN, USA). They were reconstituted in DMSO, aliquoted and stored at −20° C at a concentration of 100 µM. Just before treatment of the cells, each one of caspase inhibitors was diluted in complete DMEM at a concentration of 50 µM. Receptor antagonists SR144528 (CB2), AM251 (CB1), ML-193 (GPR-55), A784168 (TRPV1), BADGE (PPAR-γ), and MDL100907 (5-HT2A) were purchased from TOCRIS (Minneapolis, MN, USA) and were reconstituted in DMSO and stored according to manufacturers’ recommendations. DeadEnd Colorimetric TUNEL kit (Cat. no. G7360) was purchased from Promega (Madison, WI, USA). Floroblock inserts (Cat.no. 351158) were purchased from Corning (Tewksbury, MA, USA). Matrigel coated invasion chambers were purchased from VWR (Randor, PA, USA). Seahorse plates and media were purchased from Agilent Technologies (Santa Clara, CA, USA).

### Light microscopy analysis

NBL cells were seeded in a 6-well plate (Corning, Tewksbury, MA, USA) overnight to allow them to adhere to the bottom of the plate. Next day, the medium was replaced by a serum-free medium containing either DMSO (vehicle), 5 or 10 µM CBD. After 24 h, the cells were visualized for cell morphology and viability under the light microscope Olympus SZX2 stereo microscope (Center Valley, PA).

### DeadEnd™ colorimetric TUNEL system

NBL cells were plated in a 6-well plate, treated with either DMSO (vehicle) or 1 µM or 10 µM CBD in a serum-free medium for 24 h. Following manufacturer protocol, cells were washed with PBS, fixed with 4% paraformaldehyde, and permeablized with 0.2% Triton X100. Cells were then equilibrated and labelled with Terminal deoxyneocleotidyl Transferase (TdT), and the reaction stopped by blocking buffer (supplied with the kit). Cells were stained with HRP-labelled streptavidin to be visualized under bright field conditions using Cytation 5 (Bio-Tek). Dead cells that stained brown were counted by Cytation 5 software and expressed as apoptotic cell number/field.

### Annexin-PI and flow cytometry

We assessed the level of apoptosis by using the FITC Annexin-V Apoptosis Detection Kit with PI from Biolegend (San Diego, CA). Cells were plated in a 12-well plate at a density of 2.5 × 10^6^ cells/well. The following day, cells were treated for 24 h either with DMSO, 5 or 10 µM CBD in serum-free medium. Cells then were stained with Annexin-V and PI and analyzed using a FC500 Beckman Coulter flow cytometer (Indianapolis, IN, USA). For caspase inhibition, cells were treated with either DMSO (vehicle), 5 or 10 µM CBD in a serum-free medium. Cells were treated by 50 µM of caspase-2, caspase-3, caspase-8 or caspase-9 inhibitors in complete medium for one hour prior to treatment with CBD in serum-free medium. Cells then were harvested, washed, and stained for Annexin-V-PI for flow cytometry analysis.

In order to detect receptors through which CBD acts, we plated SH SY5Y cells (1 × 10^5^ cell/well) in a 24-well plate overnight. Next, cells were treated for one hour with 10 µM of AM251 (CB1 receptor antagonist), SR144528 (CB2 receptor antagonist), BADGE (PPAR-γ receptor antagonist), ML-193 (GPR-55 receptor antagonist), A784168 (TRPV1 receptor antagonist), or MDL 100907 (5-HT2A receptor antagonist) followed by 10 µM CBD in serum-free medium for 24 h. Cells then were collected, washed, and stained with AnnexinV-PI for flow cytometry analysis.

### Microarray and miRNA pathway analysis

Microarray analysis was done by using an Affymetrix array version 4.0, in order to show the integrated mature miRNA in CBD-treated cells compared to the vehicle ones, as detailed in our previous report [74]. According to manufacturer’s recommendation, 60 ng/µl of mature miRNA was purified and HSR hybridized with biotinylated Flash Tag (Affymetrix, Santa Clara, CA). The log intensity values were measured by Affymetrix system and files were analyzed using genome expression console. The files were uploaded to Transcriptome Analysis Console (TAC) from Affymetrix in order to determine miRNA fold changes and *p* values. Fold change threshold for up- or downregulated miRNA was set to ≥2 or −2 and selected for further analysis using Ingenuity Pathway Analysis (IPA) from Qiagen. The Venn diagram for the assigned miRNAs was done using the Venn diagram maker (http://bioinfogp.cnb.csic.es/tools/venny/).

### Quantitative real-time PCR (qRT-PCR)

qRT-PCR analysis was performed as detailed previously [75]. Total RNA was collected from SH SY5Y cells using the miRNeasy kit (Qiagen) per the manufacturer’s guidelines. Complementary DNA (cDNA) was reverse transcribed by using the miScript cDNA synthesis kit (Bio Rad) following the manufacturer’s recommended protocol. SSO SYBR Green (BioRad) was used on CFX context (BioRad) to perform qPCR. Human GAPDH was used as a control gene. Primers were designed in IDT DNA technologies according to the sequences found in Primers Bank (Harvard Medical School). We performed the PCR on the following protocol: 39 cycles for PCR as follows: 30 sec 98° C (denaturation step), 60 sec at 60° C (annealing step) and 60 sec at 72° C (extension step, followed by incubation for 10 minutes at 72° C.

We assessed the expression of target miRNAs and the results were normalized to Snord 96A miRNA using a SYBR Green PCR kit from Qiagen. Primer sequences are given in the following Table 1.

**Table 1:** Primer sequences for qPCR analysis of genes and miRNA in SH SY5Y cells (5′–3′)

Casp-3 forward	GAAATTGTGGAATTGATGCGTGA
Casp-3 reverse	CTACAACGATCCCCTCTGAAAAA
GAS-7 forward	CATCGCCAAGCAAAAAGCAGA
GAS-7 reverse	AGCCCAGAAGTAGTCGCAGT
BCL2L1 forward	GACTGAATCGGAGATGGAGACC
BCL2L1 reverse	GCAGTTCAAACTCGTCGCCT
SIRT2 forward	TGCGGAACTTATTCTCCCAGA
SIRT2 reverse	GAGAGCGAAAGTCGGGGAT
p53 forward	GAGGTTGGCTCTGACTGTACC
p53 reverse	TCCGTCCCAGTAGATTACCAC
MYCN forward	ACCCGGACGAAGATGACTTCT
MYCN reverse	CAGCTCGTTCTCAAGCAGCAT
PTEN forward	TTTGAAGACCATAACCCACCAC
PTEN reverse	ATTACACCAGTTCGTCCCTTTC
AKT1 forward	TCCTCCTCAAGAATGATGGCA
AKT1 reverse	GTGCGTTCGATGACAGTGGT
MDM2 forward	GAATCATCGGACTCAGGTACATC
MDM2 reverse	TCTGTCTCACTAATTGCTCTCCT
GAPDH forward	GGAGCGAGATCCCTCCAAAAT
GAPDH reverse	GGCTGTTGTCATACTTCTCATGG
Hsa-let-7a	CUAUACAAUCUACUGUCUUUC
Hsa-miR-1972	UCAGGCCAGGCACAGUGGCUCA

### miRNA- mimics and inhibitor transfections

Studies using miRNA mimics and inhibitors were performed as detailed in our previous studies [34, 76–77]. SH SY5Y cells were plated at a concentration of 4 × 10^5^ cells/well in 24-well plates overnight. The next day, cells were treated with mock, hsa-let-7a-mimic, or hsa-let-7a inhibitor for 24 h. The total RNA was collected from SH SY5Y cells using the miRNeasy kit (Qiagen) per the manufacturer’s guidelines. cDNA was synthesized using miScript cDNA synthesis kit (BioRad) followed by qPCR for genes of interest identified by Ingenuity Pathway Analysis map (IPA). After 24 h, RNA was collected for further analysis of miRNA and gene expression by qPCR as described.

### Western blot

SH SY5Y cells were plated at a density of 2.5 × 10^5^ cells/well in a 6-well plate overnight. The next day, cells were treated either with DMSO (vehicle) or CBD 10 µM in serum free medium for 6 h. Cells then were harvested, washed with PBS, and the proteinwas isolated using RIPA (radioimmunoprecipitation) lysis buffer (Santa Cruz). Twenty-five micrograms of the protein was loaded on 10% Tris-Glycine gels (Bio Rad) subjected to SDS-PAGE separation at 50V for 1 h and then at 80V for 2 h. The protein was transferred to nitrocellulose paper using iBlot2 from Invitrogen (Grand Island, NY, USA) at voltages: 20V for 1 minute, 23V for 4 minutes and 25V for 4 minutes. The membrane was blocked in 5% nonfat dry milk in TBS. The membrane was then labelled with antihuman PTEN and Akt antibodies from Santa Cruz (diluted 1:100). Secondary HRP-conjugated antibodies (Cat.no. ab6721) (Abcam) were diluted 1:1000. GAPDH (Santa Cruz) served as a reference protein diluted at 1:2000 in 5% nonfat dry milk in TBS. ECL substrate (Thermo fisher) was added to nitrocellulose paper for 3 minutes on the shaker and then photographed using an X-ray film developer. The density was measured by ImageJ from NIH.

Protein was collected from SH SY5Y cells transfected with hsa-let-7a 48 h, the level of caspase-3 protein (1:500) (Abcam), γ-tubulin (Cell Signaling) (1:2000) served as a reference protein in this experiment using the protocol described above. Secondary HRP-conjugated antibody was bought from (Abcam) and was used in a concentration of 1:2000. Cellulose membrane was stained with WesternSure Chemiluminescent substrate (LI-COR). Then, protein images were detected using C-Digit scanner (LI-COR). Image J software was used for analysis of the results, and the quantity of protein was measured after subtraction of the background by normalization to the housekeeping gene. The protein level of caspase-3 was calculated relative to corresponding mock level.

### Migration and invasion assays

Migration of NBL cells was evaluated by trans-well chambers with floro-block inserts (Corning, Tewksbury MA, USA). According to the manufacturer’s guidelines, the chambers were inserted in a 24-well cell culture plates. The cells were stained with CFDA (ThermoFisher Life Technologies, Grand Island, NY, USA). NBL cells were treated with DMSO or 10 µM CBD in a serum-free medium for 6 h. Treated cells were harvested, washed, and counted. Cells (3.5 × 10^5^) in 200 µl of serum-free medium were added to each chamber. We added 750 µl of the complete medium to the bottom of the plate. Cells were incubated for 24 h to assess cell migration through the membrane. The inserts were then washed, cells on the upper membrane were removed by swab and the remaining cells of the bottom of the insert were then visualized and counted by Cytation 5 (Bio-Tek, Winooski, VT, US).

Cell invasion was assessed using matrigel coated Corning inserts (Tewksbury MA, USA), per the manufacturer’s recommendation. Cells were plated in 24-well plates, stained with CFDA (ThermoFisher Life Technologies, Grand Island, NY, USA), washed with PBS, and treated with either DMSO or 10 µM CBD in serum-free medium for 6 h. Cells were then harvested, washed, and counted to be plated at a density of 3.5 × 10^5^ cells/well in 350 µl of the serum-free medium. 750 µl of the complete medium was added in each well. Cells were incubated for 24 h at 37° C. The inserts were washed, swabbed to remove the non-invading cells and visualized by the Cytation 5 inverted fluorescent microscope (BioTek, Winooski, VT, US).

### Metabolic assay for determination of extracellular acidification rate (ECAR) and oxygen consumption rate (OCR)

SH SY5Y cell lines were plated in 8-well Seahorse XFp Analyzer plates and allowed to adhere overnight. Cells were then treated either with DMSO (Vehicle) or 10 µM CBD in a serum-free DMEM for 6 h and are washed with XF medium per manufacturer’s recommendations. The Seahorse XFP was used to analyze the results of glycolytic stress and mitochondrial stress.

### Statistical analysis of data

Differential (upregulated or downregulated) expression of miRNAs was analyzed using 2-sample *t*-test method as described [78]. The microarray data were analyzed for significance using Kaplan-Meier method. Student’s *t*-test was used for comparing the CBD-treated group to vehicle controls. Multiple comparisons were made using ANOVA (one-way analysis of variance) test and post-hoc Tukey’s test. *p* < 0.05 was considered to be statistically significant. All experiments were repeated at least twice.

## References

[1] Colon NC, Chung DH (2011). Neuroblastoma. Adv Pediatr.

[2] Laverdiere C, Liu Q, Yasui Y, Nathan PC, Gurney JG, Stovall M, Diller LR, Cheung NK, Wolden S, Robison LL, Sklar CA (2009). Long-term outcomes in survivors of neuroblastoma: a report from the Childhood Cancer Survivor Study. J Natl Cancer Inst.

[3] Pertwee RG, Ross RA (2002). Cannabinoid receptors and their ligands. Prostaglandins Leukot Essent Fatty Acids.

[4] Iversen L (2003). Cannabis and the brain. Brain.

[5] Thomas A, Baillie GL, Phillips AM, Razdan RK, Ross RA, Pertwee RG (2007). Cannabidiol displays unexpectedly high potency as an antagonist of CB1 and CB2 receptor agonists *in vitro*. Br J Pharmacol.

[6] McKallip RJ, Lombard C, Fisher M, Martin BR, Ryu S, Grant S, Nagarkatti PS, Nagarkatti M (2002). Targeting CB2 cannabinoid receptors as a novel therapy to treat malignant lymphoblastic disease. Blood.

[7] Guzman M (2003). Cannabinoids: potential anticancer agents. Nat Rev Cancer.

[8] Ligresti A, Moriello AS, Starowicz K, Matias I, Pisanti S, De Petrocellis L, Laezza C, Portella G, Bifulco M, Di Marzo V (2006). Antitumor activity of plant cannabinoids with emphasis on the effect of cannabidiol on human breast carcinoma. J Pharmacol Exp Ther.

[9] Massi P, Vaccani A, Ceruti S, Colombo A, Abbracchio MP, Parolaro D (2004). Antitumor effects of cannabidiol, a nonpsychoactive cannabinoid, on human glioma cell lines. J Pharmacol Exp Ther.

[10] Massi P, Solinas M, Cinquina V, Parolaro D (2013). Cannabidiol as potential anticancer drug. Br J Clin Pharmacol.

[11] McKallip RJ, Jia W, Schlomer J, Warren JW, Nagarkatti PS, Nagarkatti M (2006). Cannabidiol-induced apoptosis in human leukemia cells: A novel role of cannabidiol in the regulation of p22phox and Nox4 expression. Mol Pharmacol.

[12] Calin GA, Dumitru CD, Shimizu M, Bichi R, Zupo S, Noch E, Aldler H, Rattan S, Keating M, Rai K, Rassenti L, Kipps T, Negrini M (2002). Frequent deletions and down-regulation of micro- RNA genes miR15 and miR16 at 13q14 in chronic lymphocytic leukemia. Proc Natl Acad Sci U S A.

[13] Fontana L, Fiori ME, Albini S, Cifaldi L, Giovinazzi S, Forloni M, Boldrini R, Donfrancesco A, Federici V, Giacomini P, Peschle C, Fruci D (2008). Antagomir-17-5p abolishes the growth of therapy-resistant neuroblastoma through p21 and BIM. PLoS One.

[14] Liu PY, Xu N, Malyukova A, Scarlett CJ, Sun YT, Zhang XD, Ling D, Su SP, Nelson C, Chang DK, Koach J, Tee AE, Haber M (2013). The histone deacetylase SIRT2 stabilizes Myc oncoproteins. Cell Death Differ.

[15] Sun Y, Liu PY, Scarlett CJ, Malyukova A, Liu B, Marshall GM, MacKenzie KL, Biankin AV, Liu T (2014). Histone deacetylase 5 blocks neuroblastoma cell differentiation by interacting with N-Myc. Oncogene.

[16] Fattore L, Mancini R, Acunzo M, Romano G, Lagana A, Pisanu ME, Malpicci D, Madonna G, Mallardo D, Capone M, Fulciniti F, Mazzucchelli L, Botti G (2016). miR-579-3p controls melanoma progression and resistance to target therapy. Proc Natl Acad Sci U S A.

[17] Liu S, Tetzlaff MT, Cui R, Xu X (2012). miR-200c inhibits melanoma progression and drug resistance through down-regulation of BMI-1. Am J Pathol.

[18] Scheid MP, Woodgett JR (2003). Unravelling the activation mechanisms of protein kinase B/Akt. FEBS Lett.

[19] Luo J, Manning BD, Cantley LC (2003). Targeting the PI3K-Akt pathway in human cancer: rationale and promise. Cancer Cell.

[20] Hung FC, Chao CC (2010). Knockdown of growth-arrest-specific gene 7b (gas7b) using short-hairpin RNA desensitizes neuroblastoma cells to cisplatin: Implications for preventing apoptosis of neurons. J Neurosci Res.

[21] Fisher T, Golan H, Schiby G, PriChen S, Smoum R, Moshe I, Peshes-Yaloz N, Castiel A, Waldman D, Gallily R, Mechoulam R, Toren A (2016). In vitro and *in vivo* efficacy of non-psychoactive cannabidiol in neuroblastoma. Curr Oncol.

[22] Romano B, Borrelli F, Pagano E, Cascio MG, Pertwee RG, Izzo AA (2014). Inhibition of colon carcinogenesis by a standardized Cannabis sativa extract with high content of cannabidiol. Phytomedicine.

[23] Nabissi M, Morelli MB, Amantini C, Liberati S, Santoni M, Ricci-Vitiani L, Pallini R, Santoni G (2015). Cannabidiol stimulates Aml-1a-dependent glial differentiation and inhibits glioma stem-like cells proliferation by inducing autophagy in a TRPV2-dependent manner. Int J Cancer.

[24] Morelli MB, Offidani M, Alesiani F, Discepoli G, Liberati S, Olivieri A, Santoni M, Santoni G, Leoni P, Nabissi M (2014). The effects of cannabidiol and its synergism with bortezomib in multiple myeloma cell lines. A role for transient receptor potential vanilloid type-2. Int J Cancer.

[25] Elbaz M, Nasser MW, Ravi J, Wani NA, Ahirwar DK, Zhao H, Oghumu S, Satoskar AR, Shilo K, Carson WE, Ganju RK (2015). Modulation of the tumor microenvironment and inhibition of EGF/EGFR pathway: novel anti-tumor mechanisms of Cannabidiol in breast cancer. Mol Oncol.

[26] Lombard C, Nagarkatti M, Nagarkatti PS (2005). Targeting cannabinoid receptors to treat leukemia: role of cross-talk between extrinsic and intrinsic pathways in Delta9-tetrahydrocannabinol (THC)-induced apoptosis of Jurkat cells. Leuk Res.

[27] Lukhele ST, Motadi LR (2016). Cannabidiol rather than Cannabis sativa extracts inhibit cell growth and induce apoptosis in cervical cancer cells. BMC Complement Altern Med.

[28] Chakravarti B, Ravi J, Ganju RK (2014). Cannabinoids as therapeutic agents in cancer: current status and future implications. Oncotarget.

[29] Majumder PK, Febbo PG, Bikoff R, Berger R, Xue Q, McMahon LM, Manola J, Brugarolas J, McDonnell TJ, Golub TR, Loda M, Lane HA, Sellers WR (2004). mTOR inhibition reverses Akt-dependent prostate intraepithelial neoplasia through regulation of apoptotic and HIF-1-dependent pathways. Nat Med.

[30] Marazzi J, Kleyer J, Paredes JM, Gertsch J (2011). Endocannabinoid content in fetal bovine sera - unexpected effects on mononuclear cells and osteoclastogenesis. J Immunol Methods.

[31] Opitz CA, Rimmerman N, Zhang Y, Mead LE, Yoder MC, Ingram DA, Walker JM, Rehman J (2007). Production of the endocannabinoids anandamide and 2-arachidonoylglycerol by endothelial progenitor cells. FEBS Lett.

[32] Do Y, McKallip RJ, Nagarkatti M, Nagarkatti PS (2004). Activation through cannabinoid receptors 1 and 2 on dendritic cells triggers NF-kappaB-dependent apoptosis: novel role for endogenous and exogenous cannabinoids in immunoregulation. J Immunol.

[33] Pauletto M, Milan M, Huvet A, Corporeau C, Suquet M, Planas JV, Moreira R, Figueras A, Novoa B, Patarnello T, Bargelloni L (2017). Transcriptomic features of Pecten maximus oocyte quality and maturation. PLoS One.

[34] Busbee PB, Nagarkatti M, Nagarkatti PS (2015). Natural indoles, indole-3-carbinol (I3C) and 3,3'-diindolylmethane (DIM), attenuate staphylococcal enterotoxin B-mediated liver injury by downregulating miR-31 expression and promoting caspase-2-mediated apoptosis. PLoS One.

[35] Mohan J, Gandhi AA, Bhavya BC, Rashmi R, Karunagaran D, Indu R, Santhoshkumar TR (2006). Caspase-2 triggers Bax-Bak-dependent and -independent cell death in colon cancer cells treated with resveratrol. J Biol Chem.

[36] Bouchier-Hayes L, Green DR (2012). Caspase-2: the orphan caspase. Cell Death Differ.

[37] Bisogno T, Hanus L, De Petrocellis L, Tchilibon S, Ponde DE, Brandi I, Moriello AS, Davis JB, Mechoulam R, Di Marzo V (2001). Molecular targets for cannabidiol and its synthetic analogues: effect on vanilloid VR1 receptors and on the cellular uptake and enzymatic hydrolysis of anandamide. Br J Pharmacol.

[38] Amantini C, Mosca M, Nabissi M, Lucciarini R, Caprodossi S, Arcella A, Giangaspero F, Santoni G (2007). Capsaicin-induced apoptosis of glioma cells is mediated by TRPV1 vanilloid receptor and requires p38 MAPK activation. J Neurochem.

[39] Sanchez AM, Sanchez MG, Malagarie-Cazenave S, Olea N, Diaz-Laviada I (2006). Induction of apoptosis in prostate tumor PC-3 cells and inhibition of xenograft prostate tumor growth by the vanilloid capsaicin. Apoptosis.

[40] Ryberg E, Larsson N, Sjogren S, Hjorth S, Hermansson NO, Leonova J, Elebring T, Nilsson K, Drmota T, Greasley PJ (2007). The orphan receptor GPR55 is a novel cannabinoid receptor. Br J Pharmacol.

[41] Ambros V (2001). microRNAs: tiny regulators with great potential. Cell.

[42] Zhang B, Pan X, Cobb GP, Anderson TA (2007). microRNAs as oncogenes and tumor suppressors. Dev Biol.

[43] Cheng AM, Byrom MW, Shelton J, Ford LP (2005). Antisense inhibition of human miRNAs and indications for an involvement of miRNA in cell growth and apoptosis. Nucleic Acids Res.

[44] Akao Y, Nakagawa Y, Naoe T (2006). let-7 microRNA functions as a potential growth suppressor in human colon cancer cells. Biol Pharm Bull.

[45] Dong Q, Meng P, Wang T, Qin W, Qin W, Wang F, Yuan J, Chen Z, Yang A, Wang H (2010). MicroRNA let-7a inhibits proliferation of human prostate cancer cells *in vitro* and *in vivo* by targeting E2F2 and CCND2. PLoS One.

[46] Tsang WP, Kwok TT (2008). Let-7a microRNA suppresses therapeutics-induced cancer cell death by targeting caspase-3. Apoptosis.

[47] Dent EW, Gertler FB (2003). Cytoskeletal dynamics and transport in growth cone motility and axon guidance. Neuron.

[48] Zhang Z, Zheng F, You Y, Ma Y, Lu T, Yue W, Zhang D (2016). Growth arrest specific gene 7 is associated with schizophrenia and regulates neuronal migration and morphogenesis. Mol Brain.

[49] Cimmino A, Calin GA, Fabbri M, Iorio MV, Ferracin M, Shimizu M, Wojcik SE, Aqeilan RI, Zupo S, Dono M, Rassenti L, Alder H, Volinia S (2005). miR-15 and miR-16 induce apoptosis by targeting BCL2. Proc Natl Acad Sci U S A.

[50] Tang Y, Zheng J, Sun Y, Wu Z, Liu Z, Huang G (2009). MicroRNA-1 regulates cardiomyocyte apoptosis by targeting Bcl-2. Int Heart J.

[51] Yang J, Cao Y, Sun J, Zhang Y (2010). Curcumin reduces the expression of Bcl-2 by upregulating miR-15a and miR-16 in MCF-7 cells. Med Oncol.

[52] Lai XJ, Cheng XY, Hu LD (2016). microRNA 421 induces apoptosis of c-33a cervical cancer cells via down-regulation of Bcl-xL. Genet Mol Res.

[53] Gamble LD, Kees UR, Tweddle DA, Lunec J (2012). MYCN sensitizes neuroblastoma to the MDM2-p53 antagonists Nutlin-3 and MI-63. Oncogene.

[54] Slack A, Chen Z, Tonelli R, Pule M, Hunt L, Pession A, Shohet JM (2005). The p53 regulatory gene MDM2 is a direct transcriptional target of MYCN in neuroblastoma. Proc Natl Acad Sci U S A.

[55] Bellacosa A, Kumar CC, Di Cristofano A, Testa JR (2005). Activation of AKT kinases in cancer: implications for therapeutic targeting. Adv Cancer Res.

[56] Chen L, Iraci N, Gherardi S, Gamble LD, Wood KM, Perini G, Lunec J, Tweddle DA (2010). p53 is a direct transcriptional target of MYCN in neuroblastoma. Cancer Res.

[57] Tamura M, Gu J, Matsumoto K, Aota S, Parsons R, Yamada KM (1998). Inhibition of cell migration, spreading, and focal adhesions by tumor suppressor PTEN. Science.

[58] Raftopoulou M, Etienne-Manneville S, Self A, Nicholls S, Hall A (2004). Regulation of cell migration by the C2 domain of the tumor suppressor PTEN. Science.

[59] Liliental J, Moon SY, Lesche R, Mamillapalli R, Li D, Zheng Y, Sun H, Wu H (2000). Genetic deletion of the Pten tumor suppressor gene promotes cell motility by activation of Rac1 and Cdc42 GTPases. Curr Biol.

[60] Yuan XJ, Whang YE (2002). PTEN sensitizes prostate cancer cells to death receptor-mediated and drug-induced apoptosis through a FADD-dependent pathway. Oncogene.

[61] Weng L, Brown J, Eng C (2001). PTEN induces apoptosis and cell cycle arrest through phosphoinositol-3-kinase/Akt-dependent and -independent pathways. Hum Mol Genet.

[62] Wu Y, Karas M, Dupont J, Zhao H, Toyoshima Y, Le Roith D (2004). Multiple signaling pathways are involved in the regulation of IGF-I receptor inhibition of PTEN-enhanced apoptosis. Growth Horm IGF Res.

[63] Sledzinski P, Zeyland J, Slomski R, Nowak A (2018). The current state and future perspectives of cannabinoids in cancer biology. Cancer Med.

[64] Velasco G, Sanchez C, Guzman M (2016). Anticancer mechanisms of cannabinoids. Curr Oncol.

[65] Schwartz L, Supuran CT, Alfarouk KO (2017). The Warburg Effect and the Hallmarks of Cancer. Anticancer Agents Med Chem.

[66] den Hollander B, Sundstrom M, Pelander A, Siltanen A, Ojanpera I, Mervaala E, Korpi ER, Kankuri E (2015). Mitochondrial respiratory dysfunction due to the conversion of substituted cathinones to methylbenzamides in SH-SY5Y cells. Sci Rep.

[67] DeBerardinis RJ, Lum JJ, Hatzivassiliou G, Thompson CB (2008). The biology of cancer: metabolic reprogramming fuels cell growth and proliferation. Cell Metab.

[68] Tateishi K, Iafrate AJ, Ho Q, Curry WT, Batchelor TT, Flaherty KT, Onozato ML, Lelic N, Sundaram S, Cahill DP, Chi AS, Wakimoto H (2016). Myc-Driven Glycolysis Is a Therapeutic Target in Glioblastoma. Clin Cancer Res.

[69] Ruiz-Perez MV, Medina MA, Urdiales JL, Keinanen TA, Sanchez-Jimenez F (2015). Polyamine metabolism is sensitive to glycolysis inhibition in human neuroblastoma cells. J Biol Chem.

[70] Yao A, Xiang Y, Si YR, Fan LJ, Li JP, Li H, Guo W, He HX, Liang XJ, Tan Y, Bao LY, Liao XH (2018 Oct 28). PKM2 promotes glucose metabolism through a let-7a-5p/Stat3/hnRNP-A1 regulatory feedback loop in breast cancer cells. J Cell Biochem.

[71] Nguyen LH, Zhu H (2015). Lin28 and let-7 in cell metabolism and cancer. Transl Pediatr.

[72] Arora A, Singh S, Bhatt AN, Pandey S, Sandhir R, Dwarakanath BS (2015). Interplay Between Metabolism and Oncogenic Process: Role of microRNAs. Transl Oncogenomics.

[73] Kwon OS, Han MJ, Cha HJ (2017). Suppression of SIRT2 and altered acetylation status of human pluripotent stem cells: possible link to metabolic switch during reprogramming. BMB Rep.

[74] Miranda K, Yang X, Bam M, Murphy EA, Nagarkatti PS, Nagarkatti M (2018). MicroRNA-30 modulates metabolic inflammation by regulating Notch signaling in adipose tissue macrophages. Int J Obes (Lond).

[75] Sido JM, Jackson AR, Nagarkatti PS, Nagarkatti M (2016). Marijuana-derived Delta-9-tetrahydrocannabinol suppresses Th1/Th17 cell-mediated delayed-type hypersensitivity through microRNA regulation. J Mol Med (Berl).

[76] Rao R, Rieder SA, Nagarkatti P, Nagarkatti M (2014). Staphylococcal enterotoxin B-induced microRNA-155 targets SOCS1 to promote acute inflammatory lung injury. Infect Immun.

[77] Singh NP, Miranda K, Singh UP, Nagarkatti P, Nagarkatti M (2018). Diethylstilbestrol (DES) induces autophagy in thymocytes by regulating Beclin-1 expression through epigenetic modulation. Toxicology.

[78] Singh NP, Singh UP, Nagarkatti PS, Nagarkatti M (2012). Prenatal exposure of mice to diethylstilbestrol disrupts T-cell differentiation by regulating Fas/Fas ligand expression through estrogen receptor element and nuclear factor-kappaB motifs. J Pharmacol Exp Ther.

